# A Quantitative Assessment Grading Study of Balance Performance Based on Lower Limb Dataset

**DOI:** 10.3390/s23010033

**Published:** 2022-12-20

**Authors:** Fei Wang, Anqi Dong, Kaiyu Zhang, Dexing Qian, Yinsheng Tian

**Affiliations:** AI Sports Engineering Lab., School of Sports Engineering, Beijing Sport University, 48 Xinxi Road, Beijing 100084, China

**Keywords:** balance performance, lower limb dataset, quantitative assessment model, RUS Boost, training

## Abstract

Balance ability is one of the important factors in measuring human physical fitness and a common index for evaluating sports performance. Its quality directly affects the coordination ability of human movements and plays an important role in human productive activities. In the field of sports, balance ability is an important indicator of athletes’ selection and training. How to objectively analyze balance performance becomes a problem for every non-professional sports enthusiast. Therefore, in this paper, we used a dataset of lower limb collected by inertial sensors to extract the feature parameters, then designed a RUS Boost classifier for unbalanced data whose basic classifier was SVM model to predict three classifications of balance degree, and, finally, evaluated the performance of the new classifier by comparing it with two basic classifiers (KNN, SVM). The result showed that the new classifier could be used to evaluate the balanced ability of lower limb, and performed higher than basic ones (RUS Boost: 72%; KNN: 60%; SVM: 44%). The results meant the established classification model could be used for and quantitative assessment of balance ability in initial screening and targeted training.

## 1. Introduction

Balance is one of the basic abilities of the human body to accomplish complex motor movements [1,2]. The assessment of balance in sports performance analysis is also an essential research direction in sports science today. The research results can be used not only in the rehabilitation of the elderly or people with balance disorders, but also in the selection of young athletes and the special training of various sports, which is of great value for scientific training monitoring. With the spread of wireless sensor technology in sports, physiological and sports monitoring systems consisting of multiple wearable sensors can provide coaches with a large amount of multifaceted information to provide an objective basis for training decisions [3]. Intelligent wearable equipment represented by inertial measurement unit (IMU) is now widely used and recognized by professionals in hockey, basketball, rugby, golf, gymnastics, running, swimming, etc., [4,5,6,7,8,9,10]. The speed, angle, acceleration, and other data provided by IMU can provide strong support for action posture recognition. In addition, combining motion data with supervised learning algorithms can also enable data fusion, which can be applied to comprehensive evaluation and prediction of motion performance. This method has been applied to action recognition, behavior prediction, etc., and achieved relatively good results.

The factors that affect the human balance are highly complex and, therefore, rely heavily on the expert fusion of information and comprehensive judgment. The Berg Balance Scale [11,12] was first proposed by Katherine Berg in 1989 and is the most widely used tool in balance assessment. The rehabilitator can rate the subject’s balance based on the completion of the standard action and the composite score. The balance beam test [13] and single-legged closed-eyes standing test [14] evaluate balance ability based on travel length or standing length. The test results will be observed in these tests corresponding to a performance indicator, thus alleviating the burden of complex multiple information fusion on experts, but also leading to problems such as non-uniform evaluation criteria and excessive human factor interference. The Y balance and star excursion balance tests further refine the performance metrics and synthesize them with weighting factors [15,16]. Although both methods alleviate the problem of volatility of subjective evaluation to some extent, they rely on specific testing equipment and testing movements. Therefore, they are not easily integrated with sports programs and can only be used as a reference basis. While the introduction of inertial sensor technology has greatly enriched the data sources and information dimensions, the overly complex information has also resulted in data analysis and prediction that can no longer be performed manually [17]. Various classifiers based on supervised learning algorithms can assist in the fusion of multifaceted sports data. For example, it is foreseeable that with the widespread citation of smart wearable technologies in sports, the thinking and methods of balance assessment will also be transformed. Efficient classifiers designed for various sports and special movements will be the focus of the research. However, the data used in the current classifier design are mainly from movement disorders and the elderly, and there are relatively few studies on classifiers for movement populations. In addition, since the sample classes of the motion dataset may not be balanced, classifiers designed based on traditional supervised learning algorithms such as KNN, SVM, etc., have limited performance [18].

Therefore, a three-category classifier for equilibrium prediction of the general sports population is designed based on the RUSBoost algorithm [19].

## 2. Materials and Methods

**Figure 1:**
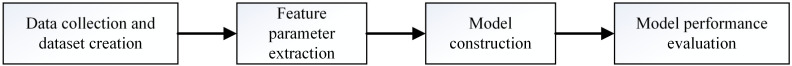
Overall process of the proposed approach.

This paper investigated the measurement method for quantitative evaluation of balance capability based on inertial measurement units, including establishing data sets through experiments, constructing quantitative evaluation models, and comparing the performance of the evaluation model and basic algorithm. The general flow of the study proposed in this paper was shown in Figure 1.

- Data collection. Including experimental movements selection using the Delphi method [20], experimental tasks designing, experimental sites setting up, and experimental data collecting. Experiments were conducted using inertial 3D motion capture system whose validity had been tested.
- Extracting key features. Principal component analysis was used to reduce the dimensionality of the features and filter out the feature parameters for classification evaluation.
- Establishing quantitative assessment models for testing balance ability. According to the characteristics of data imbalance, explored the processing methods of imbalanced data sets, and established three quantitative evaluation models of KNN, SVM, and RUS Boost.
- Model performance evaluation. Accuracy and ROC (receiver operating characteristic) curves were taken to explore the influence of different classifier algorithms on model construction, to compare and analyze the performance of models constructed by different algorithms on the test set.

### 2.1. Data Inspection

Before data acquisition, the reliability of the PNS (Perception Neuron Studio) inertial motion capture system was checked to make sure that the data collected met the acquisition standard before the formal data acquisition.

**Figure 2:**
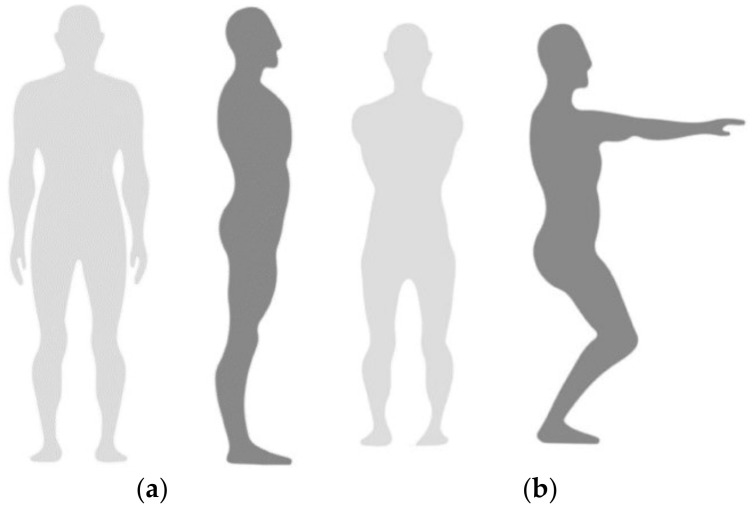
Subjects calibrate their posture. (**a**) A pose: Stand straight, arms facing down with palms facing your body. Position feet distance approximately the same distance of your hip width and maintain feet parallel to each other. (**b**) S pose: Crouch down with feet flat on the ground while maintaining feet and legs parallel to each other. Extend arms forward with palms facing down.

**Figure 3:**
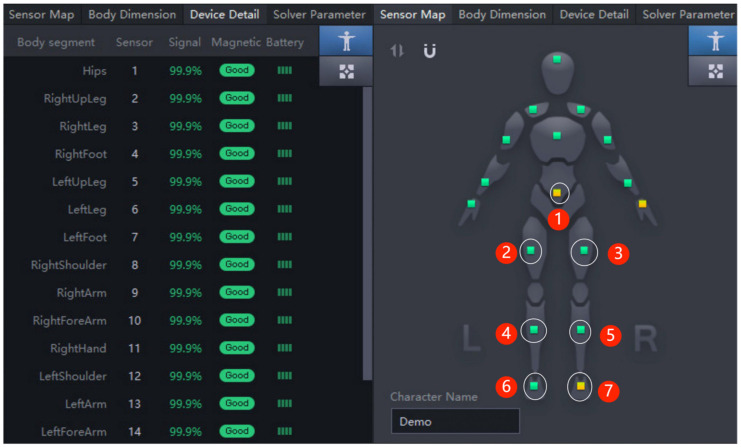
Equipment software operation interface.

The calibration procedure for this acquisition device was performed on Axis Neuron Studio software using two consecutive static poses (A pose and S pose) for four consecutive tests due to the magnetization environment during the experiment. Each pose was performed five times. During the procedure, the signal strength was observed in real time; if the signal was ‘poor’, we adjusted it in time to ensure the integrity and validity of the data. The calibration posture of the subject was shown in Figure 2, and the software interface of the device was shown in Figure 3; the seven positions marked in Figure 3 show where the lower extremity model sensors are worn.

### 2.2. Data Collection and Dataset Creation

#### 2.2.1. Experimental Pose Selection

Based on the basic concepts of balance and previous studies [21], we collected kinematic data and developed improved balance test maneuvers through literature and scales [11,22]. Based on the Delphi Action selection method, we conducted two rounds of investigation with six experts and, finally, selected four typical actions as the experimental movements for this paper. (1) Upright: this was static standing, with arms down and palms facing the body. Feet were positioned approximately the same distance as the subject’s hip width and feet are parallel to each other. (2) Walking: fixing the number of steps the subjects walked, for each subjects walking six steps. Step length should be adjusted according to the subject’s habits to prevent them from leaving the PNS reception range. (3) Squat: a 45 cm tall base was placed behind the participant’s knees; this prevented them from squatting below this depth. This standardized method was crucial to prevent reflection markers from being blocked during a lower squat. (4) BOSU ball squat [23]: the standardization of the BOSU squat was mainly from the perspective of safety, so that the subjects could maintain a consistent squat depth as far as possible under the condition of ensuring safety.

#### 2.2.2. Equipment and Environment

Shuai et al. [24] compared the lower body joint angle data obtained from both the PNS and OptiTrack systems and showed that the RMSE ranged from 3.57° to 13.14°, while the CMC values ranged from 0.47 to 0.99, indicating the reliability of the PNS in measuring lower limb kinematic data and assessing the kinematics of the lower limbs.

Data and signal acquisition equipment were mainly used to collect three-dimensional motion information from the movement of the human body. The main equipment was Perception Neuron Studio (Noitom Perception Neuron Studio, Noitom Technology Ltd., Beijing, China). According to the recommendations of the manufacture [25], the PNS IMUs were placed on the sacrum, bilaterally on the upper thigh (between the greater trochanter and medial epicondyle of the knee), the lower shank (medial surface of the proximal tibia), and the dorsum of the foot. PNS contains seven wireless IMUs, each measuring 12.5 mm × 13.1 mm × 4.3 mm and consisting of a 3-axis gyroscope (2000 DPS), 3-axis magnetometer, and 3-axis accelerometer (32 g). This system was responsible for collecting lower limb motion signals, which could be real-time transmission of acceleration (Acce), angular velocity (Gyro), quaternion (Quat), velocity (Velo), and position (Posi). In this experiment, the sampling rate of the data acquisition system was 100 Hz.

#### 2.2.3. Subjects

A total of 20 college students passed the screening for motor function and became subjects in our study. Among them, there were 10 males and 10 females with a mean age of 25.35 ± 2.35 years. Motor function screening required the subjects to perform routine functional exercise tests to ensure that the subjects had no obvious movement disorders, such as severe sports injuries, joint inflammation, or balance defects. None of the subjects in our study reported any known movement disorders or other health problems that could affect mobility. The study was conducted in accordance with the Declaration of Helsinki. Before the experiment, subjects were informed of the contents of the experiment, and each subject voluntarily signed an informed consent form and agreed to participate in the experiment.

#### 2.2.4. Experimental Procedure

The subjects wore a tight clothing with sensors on the corresponding body parts, and the system selected lower body collection mode. Before the collection, we evaluated the equipment based on the number of sensors, sensor initialization, signal quality, and sensor attitude; the equipment was used only when the collection standards were reached. To prevent the subjects from misunderstanding the actions, they should be given thorough explanations and trained on the actions before the experiment. After completing the calibration procedure, subjects would complete four rounds of testing, with each round randomly selected one pose until all poses were tested. Each pose should be completed 10 times consecutively and standard. We would record experimental videos simultaneously while collecting data and sent them to a physical training expert, who would score the poses according to her experience, with three scores: 2 (perfect), 1 (good), 0 (average). The expert scored the actions twice for evaluation, and the kappa consistency test was performed on the two scoring results. The final kappa coefficient obtained was 0.951, proving a strong intra-rater reliability. The scoring results would be used in the design of the classifier models.

#### 2.2.5. Data Statistics

The data set contained three folders IMU, BVH, and Angle. The data in the IMU folder were the original inertial CSV data exported from the test using the PNS motion capture system, and the Calc output data structure of the CSV data were shown in Table 1 [26].

The BVH folder contained the action stream BVH data [27] that define the anatomical information of the human body. BVH as a common human feature animation file format that could support most 3D animation production (3dMax, Unity, Maya) software production development [28]. BVH data were processed in the Python parsing backend of the PNS package using specific parsing methods. The action flow data format was divided into two parts: (1) SIZE: defined the size of each major bone of the body (cm) (see Table 2). (2) MOTION: defined the number of frames, the frame rate, and the rotation angle of each joint in each frame (see Table 3).

The Angle folder contained the CSV data after processing the BVH data by the Python parsing method that accompanied the PNS motion capture system. The size section clearly showed the body part position and rotation components defined in a hierarchical relationship, the motion corresponding to the skeleton information, and the data information of each frame.

### 2.3. Feature Parameter Extraction

The feature parameter extraction is mainly to extract the indicators that can characterize the motion signal. The acquired motion signal is pre-processed and analyzed in time and frequency domains, and as many time and frequency domain indicators that can reflect the motion retardation characteristics are extracted to establish the feature parameter set for motion evaluation. Feature parameters with distinguishing information, such as motion period and peak power, are extracted. Among them, mean, standard deviation, and RMS are commonly used feature parameters in the field of balance quantitative assessment studies. For each movement, the extracted feature parameters are 35 features such as acceleration root mean square, angular velocity root mean square, combined acceleration, combined angular velocity, approximate entropy (ApEn), principal frequency, total energy value, coefficient of variation, mean motion period, and peak power, among other features [29].

The extracted feature values were subjected to feature dimensionality reduction using principal component analysis, and the Bartlett ball test could determine whether the factor analysis method was effective for the extracted features. If the Bartlett’s spherical test significance of the extracted action feature parameters was less than 0.05, indicating that the principal component factor analysis method was valid [30].

**Figure 4:**
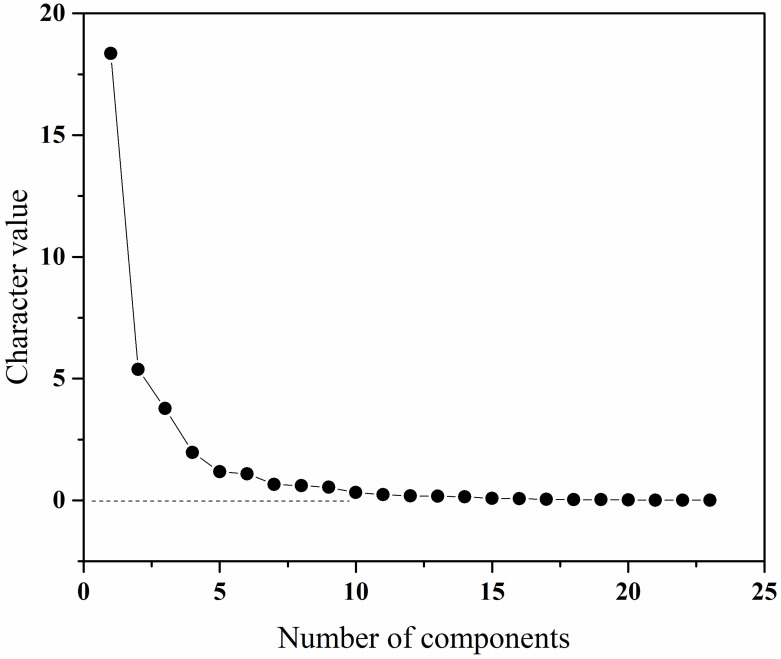
Gravel plot of the explanatory rate of the characteristic variables.

As shown in Figure 4, it could be seen that the gravel plot tends to flatten out after 12 factors, which could indicate that the explanation rate of the variables for the 12 factors extracted from the derived actions reaches more than 98%, so for each action feature the 12 factors extracted could be used as the feature parameters for placement into the evaluation model.

Finally, 12 reserved feature parameters of the placement assessment model were shown in Table 4.

### 2.4. Model Construction

**Figure 5:**
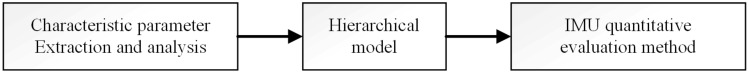
Flow chart of quantitative assessment model building.

In machine learning, common classification algorithms included SVM, decision tree, KNN, BP neural network, plain Bayesian, and integration learning methods [31]. For the same dataset, each classification method had different performance. In this paper, we compared and validated three common classification algorithms, such as KNN classifier, support vector machine, and RUS Boost classifier, and compared the recognition accuracy of evaluation grading models constructed by different classification algorithms on the test set to construct a grading evaluation model for quantitative balancing ability. Figure 5 showed the flow chart of quantitative assessment model building. 

**Figure 6:**
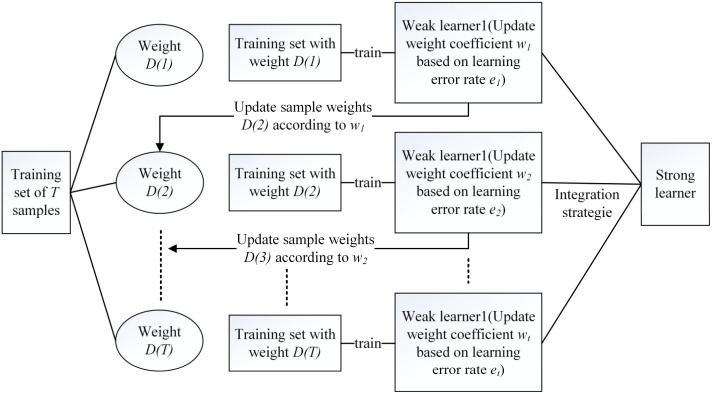
RUS Boost basic logic diagram.

Integrated learning algorithms, in general, consist of a series of basic classifiers. The two main integrated learning algorithms were Boosting and Bagging. Two mainstream integration learning algorithms were Boosting and Bagging, which were widely used in classification studies of unbalanced datasets due to their good performance in learning classification of unbalanced datasets by resampling the training data or improving the classifier algorithm. When there were far more samples in one class than in other classes, a classifier that could effectively identify samples from underrepresented classes needed to be constructed [19,32]. RUS Boost was a classification algorithm based on boosting for the class imbalance problem in class-labeled data, and its basic logic was shown in Figure 6.

**Figure 7:**
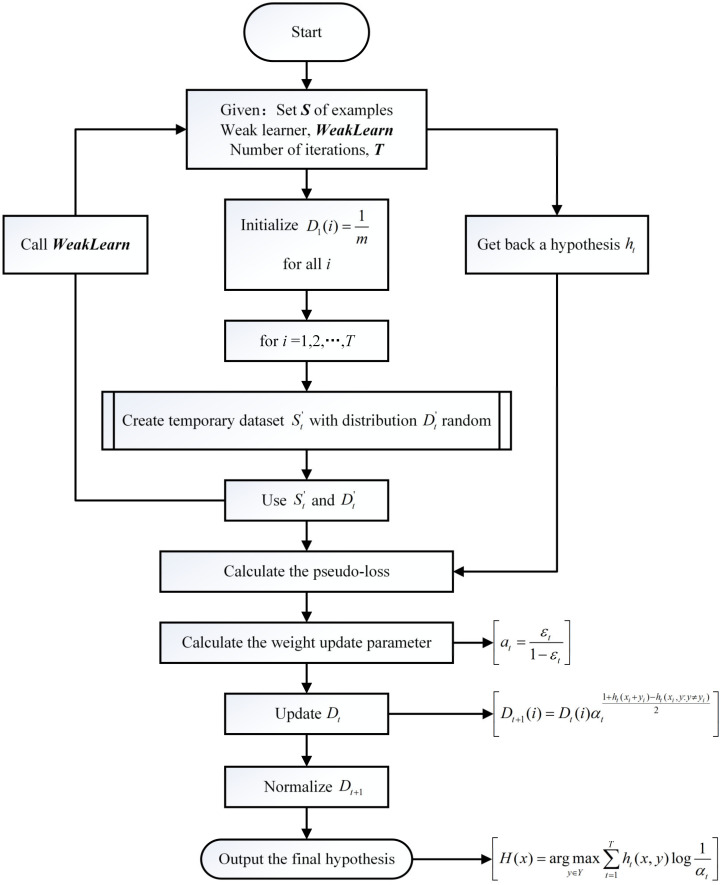
Flow chart of RUS boost basic steps.

The RUS Boost algorithm introduced the random under sampling technique into the algorithm of AdaBoost, combining the data sampling technique and the Boosting algorithm, which was not only fast and efficient, but also had an excellent performance in the classification of unbalanced datasets [33]. In this paper, the RUS Boost algorithm was used to adjust the classification of the dataset to avoid the impact of the weak classification ability of a single classifier and to improve the classification ability of the unbalanced dataset. Figure 7 showed the diagram of the basic steps of RUS Boost.

**Figure 8:**
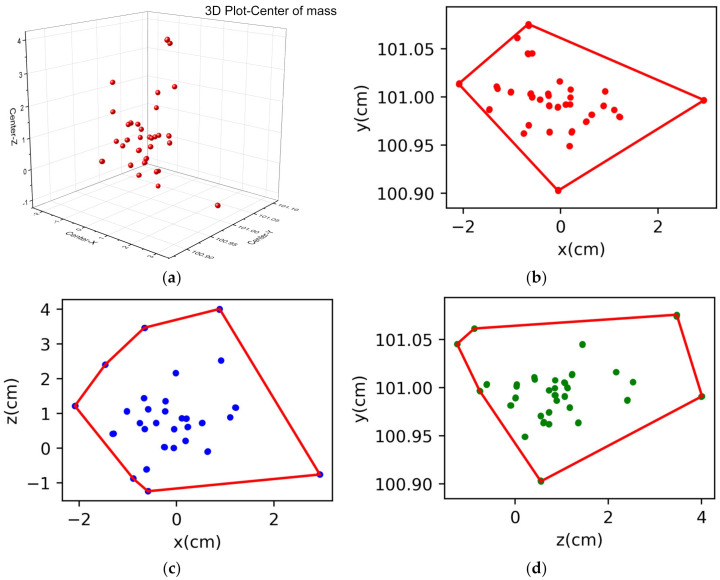
(**a**) The 3D scatter of the mass center of the last three frames. (**b**) The convex hull graph distribution of projection on the XY axis. (**c**) The convex hull graph distribution of projection on the XZ axis. (**d**) The convex hull graph distribution of projection on the YZ axis.

After calibrating all sensors, the initial posture of the subject was aligned with the posture of the models in the software, as shown in Figure 1, with the face forward and the arms hanging naturally on the sides of the subject. All models were kept in the center area of the software sensor area to keep the sensor positions of the models stable. To evaluate the quality of the calibrated data, the X, Y, and Z positions of the hip, knee, and ankle joints and their posture angles in the first frame were extracted from each calibration data record using the PNS software, and the data distributions of the three dimensions were relatively concentrated, indicating that our calibration method was effective. The results of the data distribution are shown in Figure 8.

## 3. Results

### 3.1. Comparison of Quantitative Model Performance

In this paper, confusion matrix, ROC curve, AUC, and accuracy were used to compare and analyze the strengths and weaknesses of the quantitative models constructed using three classification algorithms on the test set, and the performance of the graded models were respectively compared for KNN classification algorithm, SVM support vector machine classification algorithm, and RUS Boost integrated algorithm. According to the balance degree mentioned in Section 3.1, there are three levels: 2, excellent; 1, good; 0, general.

#### 3.1.1. KNN

**Figure 9:**
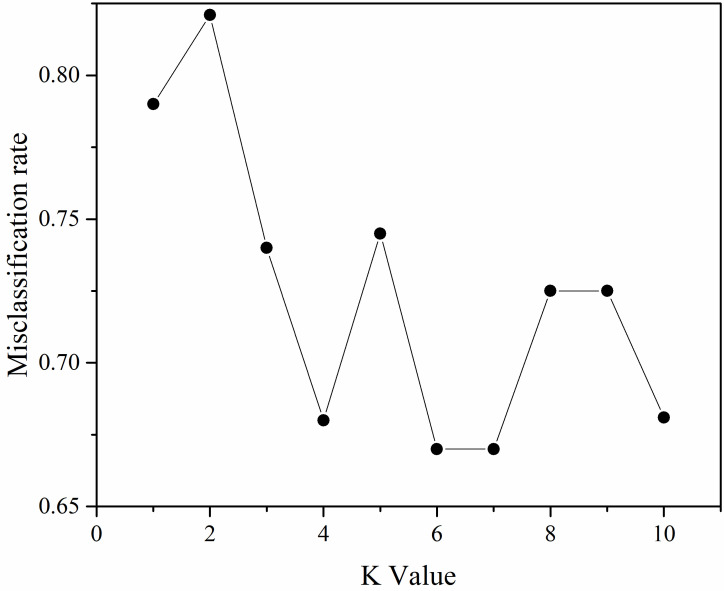
KNN model K-value selection.

**Figure 10:**
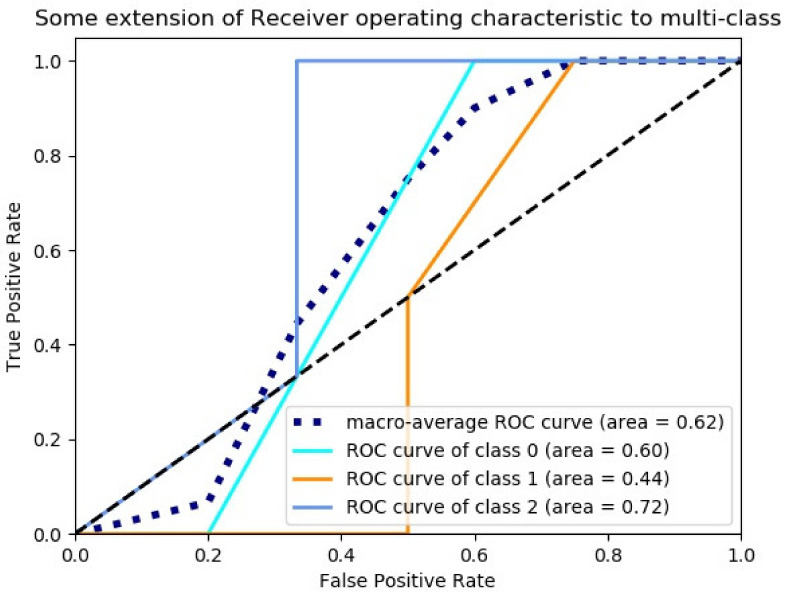
KNN classification model ROC curve and area under the ROC curve.

After analysis, it was concluded that the error value was minimum when K = 6 or 7 as shown in Figure 9, but considering that K generally is odd number, therefore, K = 7 was finally chosen. The KNN classification results were shown in Figure 10.

#### 3.1.2. SVM

**Figure 11:**
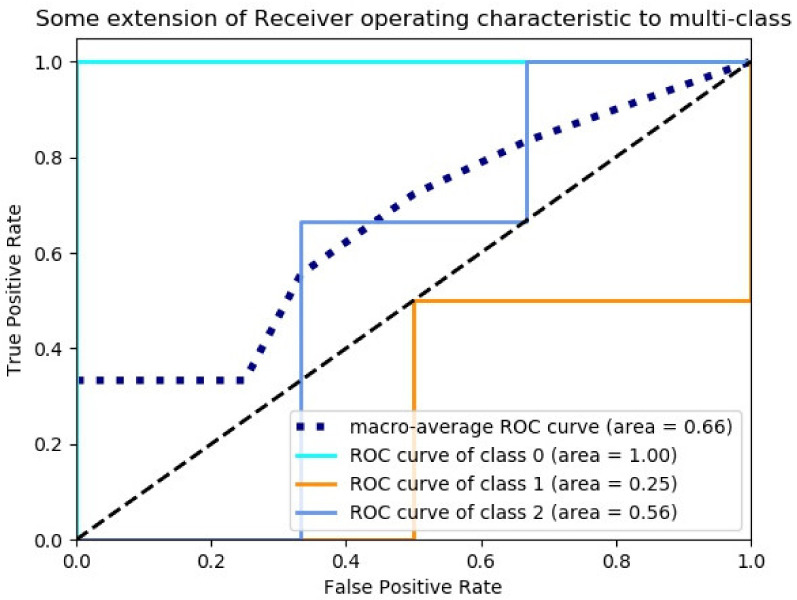
SVM classification model ROC curve and area under the ROC curve.

Support vector machine: the penalty function parameter C was set to 0.5, and the classification results were shown in Figure 11.

#### 3.1.3. RUS Boost

**Figure 12:**
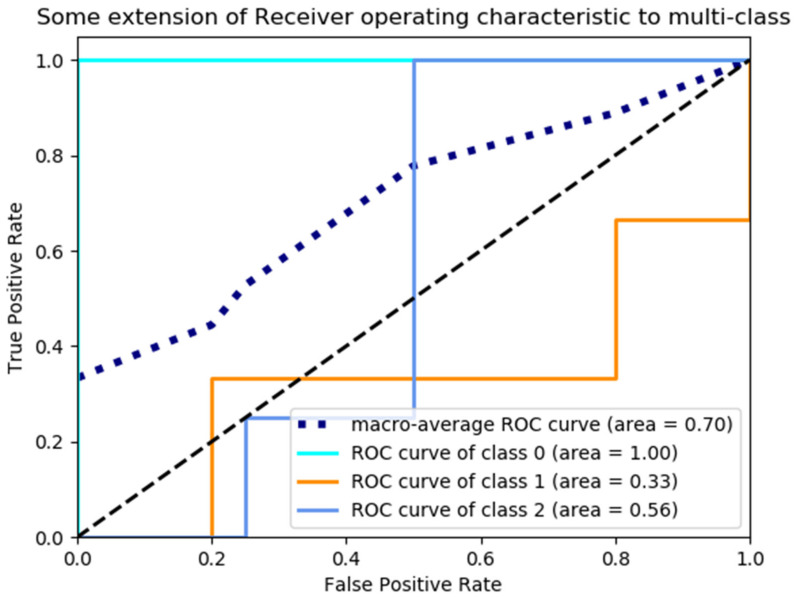
RUS classification model ROC curve and area under the ROC curve.

The base iterative classifier was selected as SVM, the total number of iterations was set to 100, and the learning rate was 0.2. The classification results were shown in Figure 12.

## 4. Discussion

Prior studies have established a number of validated balance tests such as Berg scales [11], Y-balance tests [15], etc. In this study, we proposed a grading method for quantitative assessment of balance ability based on inertial sensors, and established a grading model for balance ability assessment by feature extraction and analysis of the dataset, and then compared the performance of the grading assessment model.

### 4.1. Classifier Performance

KNN classification algorithm is one of the simplest methods in data mining classification techniques, which is robust, general and simple to understand [34]. It could be obtained from the above data and classification results that KNN had better performance in detecting triple classification compared to other models, with an over-all evaluated accuracy of 0.62. The parameter C in SVM is the penalty parameter, which represents the misclassification or error term. If the penalty parameter is set higher, the error rate of classification is smaller, but if the penalty parameter is set too high when using a nonlinear kernel it may cause overfitting [35]. Therefore, setting the penalty parameter of the SVM to 0.2, the accuracy of SVM is 0.66. 

Previous research has used the ensemble method to address problems in cybersecurity, fraud detection, recommender systems, healthcare, and remote sensing [36].Therefore, SVM was used as the base classifier in RUS Boost classification, and the RUS Boost algorithm was a cumulative prediction of multiple SVM models, and the algorithm. The final RUS Boost classification accuracy, which was 0.70, had been improved due to the cumulative prediction using a single classification model, indicating that this type of integrated prediction method was generally better than a single classification model. Most of the traditional machine learning algorithms assume that the data distribution is uniform, and the imbalance of data distribution will affect the performance of the classifier to some extent, which is likely to be the reason for the poor classification results of the previous classes of algorithms. The RUS Boost algorithm combines the undersampling technique with Boosting, which balances the original data set by reducing the samples of most classes, although a certain amount of information is lost, but since the Boosting algorithm constructs different weak classifiers, the amount of information lost in one weak classifier is likely to be included in other weak classifiers [19,37]. Therefore, RUS Boost algorithm has a great advantage in classifying unbalanced data, which is also demonstrated in this study.

The classification accuracy of the scoring model constructed by the RUS Boost algorithm was proved to be 70%, which can meet the expectation well and can be used to some extent as a supplement to the scale to evaluate the human balance ability.

### 4.2. Limitation and Future Work

A number of limitations need to be noted regarding the present study. First, all participants in this study were healthy adults, and further studies are needed to verify whether the study is reliable in pathological populations or in elite athletes.

Second, a large number of data samples are missing. The quantitative model based on machine learning can be more accurate and objective under the condition of sufficient data. Further work is required to establish the viability of expanding the sample size and further optimizing the classifier to build the system.

Third, although the currently used classifier could further improve the imbalance of the data, the subsequent superposition of the data volume would lead to an increasing rate of misclassification, and the classification accuracy could be effectively improved by changing the number of iterations and further processing of the data.

## 5. Conclusions

In this study, the lower limb dataset was analyzed for feature extraction using factor analysis with principal component analysis, and the final extracted 12 factors were obtained as the features extracted after dimensionality reduction, and KNN, SVM, and RUS Boost classification models for the unbalanced dataset were proposed and evaluated for performance. The performance comparison yielded a better performance of the RUS Boost algorithm for classification in balanced quantization, with a detection accuracy of 70%. The experimental results showed that the RUS Boost classification algorithm performs better and provided a better quantitative assessment of the balancing ability because the data obtained from the experiments are unbalanced datasets. The classifier may be able to replace the assessment scale to a certain extent to evaluate the balance ability.

## Figures and Tables

**Table 1:** Calc data structure table.

Serial Number	Marking	Content Notes
1	Body part name	A total of 7 species, see Table 2 for details
2	Characteristic information	Sensor, Joint, Bone
3	Physical quantity information	Transmission of acceleration (Acce), angular velocity (Gyro), quaternion (Quat) and velocity (Velo) and position (Posi)
4	Specific component values	x, y, z components, where the quaternion has one more w component

**Table 2:** Table of body part names *.

Partial Name	Marking	Serial	Parent Node
Hip	Hips	0	Root Node
Right thigh	RightUpLeg	1	0
Right calf	RightLeg	2	1
Right foot	RightRoot	3	2
Left thigh	LeftUpLeg	4	3
Left calf	LeftLeg	5	4
Left foot	LeftFoot	6	5

* In the data, the lower limbs are divided into 7 parts, where the hip node is the root node and each subsequent node is connected to a parent node in the order shown in the table above.

**Table 3:** Bone and joint correspondence table.

Bone	Joint
The connection between Hip and LeftUpLeg	LeftHip
The connection between LeftUpLeg and LeftLowLeg	LeftKnee
The connection between LeftLowLeg and LeftFoot	LeftAnkle
The connection between Hip and RightUpLeg	RightHip
The connection between RightUpLeg and RightLowLeg	RightKnee
RightFoot	RightAnkle

**Table 4:** Explanation of main characteristic parameters.

Serial Number	Characteristic Quantity Name	Specific Meaning
1	KU_SA	Combined acceleration kurtosis
2	SK_SW	Combined angular velocity skewness
3	SK_SA	Combined acceleration skewness
4	KU_VY	Y-axis velocity kurtosis
5	RMS_SW	Root mean square of the combined angular velocity
6	KU_SW	Combined angular velocity skewness
7	RMS_SA	Root mean square of the combined acceleration
8	SK_AY	Y-axis acceleration skew
9	KU_AZ	Z-axis acceleration kurtosis
10	RMS_AZ	Root mean square of Z-axis acceleration
11	KU_AY	Y-axis acceleration kurtosis
12	ME	Average total energy

## Data Availability

The dataset mentioned in this study can be found at https://doi.org/10.6084/m9.figshare.20579541.v1 (accessed on 1 September 2022).
